# Complete Genome Sequence of the Strain of *Lymantria dispar* Multiple Nucleopolyhedrovirus Found in the Gypsy Moth Biopesticide Virin-ENSh

**DOI:** 10.1128/genomeA.01407-14

**Published:** 2015-01-15

**Authors:** Robert L. Harrison, Daniel L. Rowley

**Affiliations:** U.S. Department of Agriculture, ARS Invasive Insect Biocontrol and Behavior Laboratory, Beltsville, Maryland, USA

## Abstract

We report the genome sequence of an alphabaculovirus from the gypsy moth (Lymantria dispar) biopesticide Virin-ENSh. The genome sequence is 161,712 bp, and its structure and sequence similarity indicate that the virus used in Virin-ENSh is a strain of the species *Lymantria dispar* multiple nucleopolyhedrovirus.

## GENOME ANNOUNCEMENT

The gypsy moth, Lymantria dispar (Lepidoptera: Noctuidae), is a worldwide pest of trees and forests (1). Research on baculoviruses isolated from gypsy moth larvae resulted in the development of baculovirus-based biopesticides for L. dispar control, most notably Gypchek in the United States (2) and Virin-ENSh in the former Soviet Union (3). Further studies with the Gypchek virus strain LDP-67 and with plaque isolates derived from LDP-67 indicated that the virus in Gypchek is a group II nucleopolyhedrovirus of genus *Alphabaculovirus* (family Baculoviridae), designated the species Lymantria dispar multiple nucleopolyhedrovirus (LdMNPV) (4). The complete genome sequence for LdMNPV 5-6, a plaque isolate derived from LDP-67, serves as the reference genome sequence for this species (5). Partial sequencing of three loci from the Virin-ENSh virus suggested that the LDP-67 and Virin-ENSh viruses are isolates of the same baculovirus species (6). However, it has been claimed that the Virin-ENSh virus was originally isolated from a different host species (7), and restriction endonuclease digests of LDP-67 and Virin-ENSh DNA yielded significantly different fragment patterns when analyzed by agarose gel electrophoresis (8).

To resolve issues surrounding the identity of the virus found in Virin-ENSh, occlusion bodies of a sample of Virin-ENSh isolate 3029 of a USDA insect virus collection maintained in Beltsville, Maryland, USA, were grown in New Jersey Standard Strain gypsy moth larvae. Viral DNA was isolated from the occlusion bodies and subjected to 454 sequencing as previously described (6). Assembly of the sequencing data resulted in a final contig of 161,712 bp with a coverage of 133×. In comparison with the LdMNPV 5-6 genome sequence, the Virin-ENSh/LdMNPV-3029 genome sequence exhibited 98.2% nucleotide sequence identity and contained 154 of the 163 open reading frames (ORFs) annotated for LdMNPV 5-6. However, the pairwise alignment between these two sequences included 410 gaps covering a total gap length of 8,607 bp. The gaps tended to be concentrated in regions of the genomes containing repeated sequence elements, particularly homologous repeat regions (*hr*s) and baculovirus repeated ORFs (*bro*s). The two LdMNPV genomes contain 12 (LdMNPV-3029) or 13 (LdMNPV 5-6) *hr*s and 16 *bro*s found in 8 clusters distributed throughout the genome sequences. Large indels occurring in these locations provide a likely explanation for the appearance of significantly different restriction endonuclease fragment patterns reported for these two strains of LdMNPV. We conclude that the virus used in the Virin-ENSh biopesticide is a strain of the previously defined baculovirus species *Lymantria dispar* multiple nucleopolyhedrovirus.

### Nucleotide sequence accession number.

The genome sequence was deposited in GenBank under the accession number KM386655.

## References

[1] Pogue MG, Schaefer PW 2007 A review of selected species of Lymantria (Hubner [1819]) (*Lepidoptera*: *Noctuidae*: *Lymantriinae*) from subtropical and temperate regions of Asia, including the descriptions of three new species, some potentially invasive to North America, no. FHTET-2006-7. Forest Health Technology Enterprise Team, Forest Service, U.S. Department of Agriculture, Fort Collins, CO.

[2] Reardon RC, Podgwaite J, Zerillo R 2012 Gypchek—environmentally safe insecticide for gypsy moth control, no. FHTET-2012-01. Forest Health Technology Enterprise Team, Forest Service, U.S. Department of Agriculture, Morgantown, WV.

[3] Alyoshina OA 1980 Study of entomopathogenic viruses in the USSR, p 1–16. In Ignoffo CM, Martignoni ME, Vaughn JL (ed), Characterization, production and utilization of entomopathogenic viruses. Proceedings of the Second Conference of Project V, Microbiological Control of Insect Pests of the US/USSR Clearwater Beach, Florida, USA.

[4] Herniou EA, Arif BM, Becnel JJ, Blissard GW, Bonning B, Harrison RL, Jehle JA, Theilmann DA, Vlak JM 2011 Baculoviridae, p 163–174. In King AMQ, Adams MJ, Carstens EB, Lefkowitz EJ (ed), Virus taxonomy: ninth report of the international committee on taxonomy of viruses. Elsevier, Oxford.

[5] Kuzio J, Pearson MN, Harwood SH, Funk CJ, Evans JT, Slavicek JM, Rohrmann GF 1999 Sequence and analysis of the genome of a baculovirus pathogenic for *Lymantria dispar*. Virology 253:17–34. doi:10.1006/viro.1998.9469. 9887315

[6] Harrison RL, Keena MA, Rowley DL 2014 Classification, genetic variation and pathogenicity of *Lymantria dispar* nucleopolyhedrovirus isolates from Asia, Europe, and North America. J Invertebr Pathol 116:27–35. doi:10.1016/j.jip.2013.12.005. 24370838

[7] Lipa JJ 1998 Eastern Europe and the former Soviet Union, p 216–231. *In* Hunter-Fujita F, Entwhistle PF, Evans HF, Crook NE (ed), Insect viruses and pest management. John Wiley & Sons, Chichester, United Kingdom.

[8] Dougherty E 1983 A comparison of the Gypchek and Virin-ENSh preparations of a multiple embedded nuclear polyhedrosis virus of the gypsy moth, *Lymantria dispar* utilizing restriction endonuclease analysis, p 21–30. In Ignoffo C, Martignoni ME, Vaughn JL (ed), A comparison of the US (Gypchek) and the USSR (Virin-Ensh) preparations of the nuclear polyhedrosis virus of the gypsy moth, *Lymantria dispar*: results of research conducted under project V-01.0705, Microbiological Control of Insect Pests of the US/USSR Joint Working Group on the Production of Substances by Microbiological Means. American Society for Microbiology, Washington, DC.

